# The differential effects of type and frequency of social participation on IADL declines of older people

**DOI:** 10.1371/journal.pone.0207426

**Published:** 2018-11-21

**Authors:** Kimiko Tomioka, Norio Kurumatani, Keigo Saeki

**Affiliations:** Nara Prefectural Health Research Center, Nara Medical University, Kashihara, Japan; Banner Alzheimer's Institute, UNITED STATES

## Abstract

**Background:**

Although social participation (SP) is valid in active aging, it is vague which types and the frequency of SP are effective in maintaining instrumental activities of daily living (IADL). We conducted a community-based prospective cohort study and investigated the association of the types and frequency for SP with IADL decline in community-dwelling older adults.

**Methods:**

The target population were all individuals aged ≥65 living in a commuter town in Nara, Japan. A total of 6,013 participants with independent IADL at baseline were analyzed. IADL was assessed using the Tokyo Metropolitan Institute of Gerontology Index of Competence. Six SP types were assessed: volunteer groups, sports groups, hobby clubs, senior citizens’ clubs, neighborhood community associations, and cultural clubs. The frequency of SP was categorized into frequent (i.e., weekly or more), moderate (i.e., monthly or yearly), and non-participation. Using multiple logistic regression models, the odds ratio (OR) and a 95% confidence interval (CI) for IADL decline were calculated. Covariates included age, marital status, education, subjective economic status, work status, body mass index, chronic medical conditions (i.e., hypertension, diabetes mellitus, heart disease, and cerebrovascular disease), lifestyle factors (i.e., alcohol, smoking, and exercise), self-rated health, depression, and cognitive functioning. To examine gender differences, stratified analyses by gender were performed.

**Results:**

During the 33-month follow-up, 16.4% of men and 8.7% of women exhibited IADL decline. After adjustment for all covariates, compared to those who never participated, women with moderate participation had significantly lower odds of IADL decline in volunteer groups (OR = 0.53, 95% CI = 0.31–0.88), hobby clubs (OR = 0.55, 95% CI = 0.38–0.79), neighborhood community associations (OR = 0.58, 95% CI = 0.42–0.81), and cultural clubs (OR = 0.51, 95% CI = 0.31–0.82), and women with frequent participation had lower odds of IADL decline in hobby clubs (OR = 0.63, 95% CI = 0.43–0.93). In contrast, among men, the significant association between SP and less risk of IADL decline was limited to moderate participation in neighborhood community associations (OR = 0.79, 95% CI = 0.63–0.99), and there were no differences between frequent participation and non-participation in all types of SP. Regarding volunteer groups, compared to women with frequent participation, women with moderate participation had a significantly lower risk of IADL decline (OR = 0.37, 95% CI = 0.18–0.77). The results of additional stratified analyses by self-rated health, depression, and cognitive functioning showed that the associations of the type and frequency of SP with IADL decline varied according to physical and mental functioning.

**Conclusions:**

Several types of SP have a favorable effect on IADL through moderate participation rather than frequent participation, and women with moderate participation in volunteer groups have a more beneficial effect on IADL than women with frequent participation. When advising community-dwelling older adults on SP for IADL maintenance, health professionals may need to take into account plateau effects, gender differences, and physical and mental functioning.

## Introduction

Nowadays, the rising proportion of older people in the population is a worldwide issue, and the promotion of a healthy aging society is vital for both advanced and emerging nations [1]. Considered a recipe for extending healthy life expectancy, social participation (SP) has gained great attention [2]. The following is proposed as the mechanism between greater SP and positive health effects for older people. Because SP can bring about interaction with others in the community [3], older adults who participate in social activities tend to feel fulfilled, avoid social withdrawal and loneliness, have access to information about health promotion, and be able to cope with stress-related harmful impacts on mind and body, compared to those who have no SP [4–6]. Although there are a multitude of prior studies reporting that SP in older age can positively affect their health [7–20], some studies have suggested that more frequent SP does not necessarily mean that older adults obtain more health benefits. For example, Musick et al. reported that volunteering in moderate amounts was associated with longevity, while frequent volunteering did not differ from not volunteering at all [17]. Takeuchi et al. found that the positive association between dental health and participation in community activities was observed in older adults with yearly participation, but not in those with monthly or weekly participation [19]. Although these studies are open to debate because of limits in type of SP [17] or due to being a cross-sectional study [19], they have demonstrated that health benefits of SP may have a plateau effect. Furthermore, some researchers have indicated that the associations of SP with older adults’ health vary in accordance with the type of SP [7,9,14,19,20] and the gender of participants [7,8,11,12,16,20]. Regarding the type of SP, Japanese prior studies reported that parents’ and teachers’ associations [12] and neighborhood community associations [19] were not associated with better health, and pointed out that the obligatory participation characteristic of these SP types may negate its health benefits. Regarding gender difference, according to the role strain hypothesis [5], because SP tends to place a bigger emotional burden on women than on men, women may have fewer health advantages from SP than men. In contrast, based on social role hypothesis [21], because men are less likely to engage in domestic activities than women, older men can have more time to participate in activities outside of the home, which in turn have greater SP effects than those for older women. Some studies reported that greater SP was associated with a greater reduction in health risk for men than for women [11,16], while other studies found that women gained more health benefits from SP than men [7,12,20], or that there were no gender differences in health consequences by SP [8,9]. Thus, the association between SP and older adults’ health should be investigated separately for men and women, based on the type and frequency of SP.

Based on the main health index of older adults, many studies have adopted mortality [8,11,17], disability in basic activities of daily living [9,10,13–15], self-rated health (SRH) [12,16], depression [18], and cognitive functioning [20] as outcome measures. However, in relation to active aging, higher-level functional capacity is a more suitable indicator for prolonging healthy life expectancy, because it requires a level of competence to live independently in the community [22]. In particular, instrumental activities of daily living (IADL) relate to the ability to look after oneself, for example, preparing meals, using public transport, managing money and medication. Essentially, maintaining IADL means enjoying longevity in a state of being self-supporting. For this reason, we considered IADL as a vital health indicator for older adults, and have conducted research focusing on the association between IADL and SP from the viewpoint of the type and frequency of SP according to gender [23,24]. Although our previous studies reported that the positive association between frequent SP and IADL was greater among older females than older males, causal relationships were unconfirmed due to cross-sectional design. Additionally, we conducted a community-based longitudinal study of the association between SP and IADL [25], but this study failed to evaluate the frequency of SP.

The current study’s goal, therefore, is to verify the association between the types and frequency of SP and IADL through a prospective cohort study in community-dwelling older adults. Our points of interest were the following: which type of SP was effective in IADL maintenance, whether more frequent SP offered more benefits in preventing IADL decline, and whether the relationship between SP and IADL differed in men and women.

## Methods

### Study population

The details of this cohort study are explained elsewhere [26]. Briefly, we conducted a baseline survey in March 2014 and a follow-up survey in November 2016. Self-administered questionnaires were distributed to all people who satisfied the following two conditions: 1) living in a commuter town in Nara Prefecture in Japan, and 2) aged ≥65 as of January 1, 2014. The exclusion criteria were: 1) people living in a nursing home or people in hospital at survey, 2) people with IADL dependence at baseline, and/or 3) people with missing data regarding IADL and/or SP.

**Fig 1:**
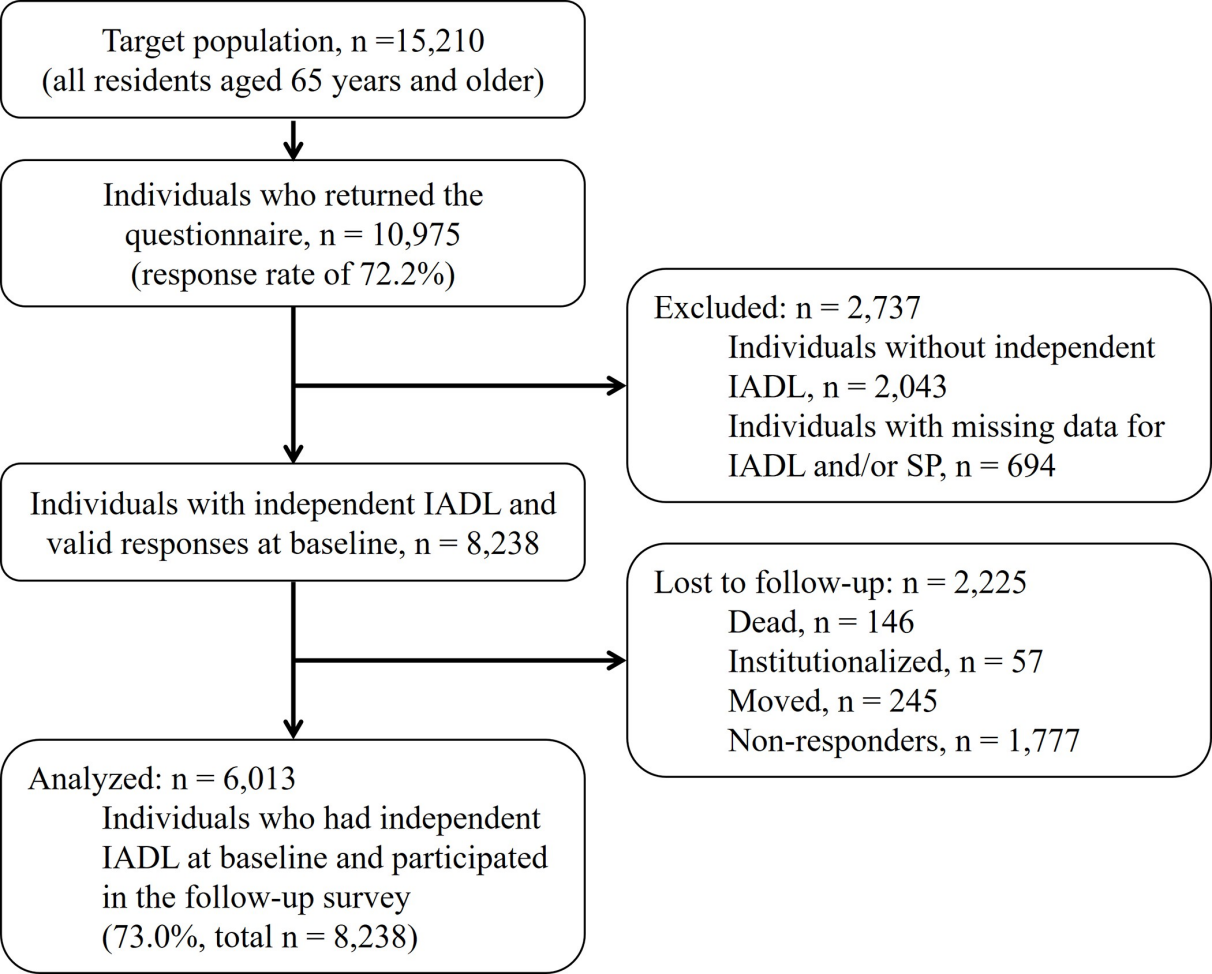
Flow diagram of participant enrollment. IADL, instrumental activity of daily living; SP, social participation.

Fig 1 displays a flow diagram of participant enrollment. In March 2014, the city office mailed the baseline questionnaires to 15,210 community-dwelling older adults. Of these, 10,975 (72.2%) participated in the baseline survey. We excluded 2,737 persons from the follow-up survey because of dependent IADL at baseline (n = 2,043) or missing data for IADL and/or SP (n = 694). We followed the 8,238 persons who had independent IADL and valid responses at baseline until November 2016, and gathered follow-up data on new-onset IADL decline. We failed to obtain follow-up data for 2,225 persons because of death (n = 146), nursing home admissions (n = 57), migration (n = 245), or non-response (n = 1,777). Thus, we included 6,013 persons in the final analyses (mean age ± SD: 72.8 ± 5.8; age range: 65–96; 43.9% male).

Compared to persons included in the final analyses (i.e., analyzed individuals), persons excluded because of missing data or loss to follow-up were more likely to be older and have poorer SRH, more depressive symptoms, and poorer cognitive functioning. There was no difference in gender between the analyzed and excluded groups (data shown in S1 Table).

This study was approved by the Nara Medical University Ethics Committee (approval numbers 939). All study participants provided signed informed consent.

### Assessment of social participation

Based on prior studies [9,10,13,14,19,23–25], SP was classified into six types: volunteer groups, sports groups, hobby clubs, senior citizens’ clubs, neighborhood community associations, and cultural clubs. Respondents were asked about their frequency of participation in each activity: ≥4 times a week, several times a week, once a week, several times a month, several times a year, or never. Prior studies have suggested that people who participate in activities on a weekly basis are frequent participants and people who participate in activities on a monthly or yearly basis are moderate participants [13,15,17,27,28]. Therefore, in this study, SP was classified into three categories: frequent = not less than once a week (i.e., weekly or more), moderate = several times or less a month (i.e., monthly or yearly), and non-participation.

The types of SP that are unique to Japan are senior citizens’ clubs and neighborhood community associations [28]. Senior citizens’ clubs are for older people who live in the community. This club has an age limit (i.e., club members are composed of people aged 60 or older). Japanese senior citizens’ clubs are supported by the national or local government. They conduct a variety of activities for older people to prevent decline in physical and mental functioning. Activities include sports, mental exercise, and performing arts. Neighborhood community associations serve as community organizations in the public domain, which perform a broad range of activities, such as community festivals or events, environmental beautification, disaster prevention, crime prevention, and home visit for older adults living alone. Basically, participation in neighborhood community associations is voluntary. However, some communities have a participation rate of around 100%, which suggest that neighborhood community associations contain an element of mandatory participation.

### Assessment of instrumental activities of daily living (IADL)

IADL was assessed using 5 items from the Tokyo Metropolitan Institute of Gerontology Index of Competence (TMIG-IC) [29], based on a subjective evaluation by the respondents themselves. The TMIG-IC was developed to measure higher-level functional capacity among older adults who are living in the community, and has been commonly used in Japan. The validity and reliability of the TMIG-IC were ascertained in a prior study [30]. Respondents were asked whether they could perform five activities: use of public transport, shopping, meal preparation, bill payment, and money management. Scores range from 0 to 5; higher scores show higher level in IADL performance. Respondents who answered “able to perform” in all of five activities were given full marks of 5 points, and classified as persons with independent IADL [31]. Respondents unable to perform one or more activity were classified as those with dependency in IADL. We followed up only people with independent IADL at baseline for 33 months. Decline in IADL was defined as a decrement of 1 or more points (i.e., IADL score was 4 and below at a 33-month follow-up). This cut-off value is based on research carried out to assess test-retest variation in the TMIG-IC; the variation of 1 point for IADL score is taken as a meaningful change in IADL performance [32].

### Covariates

Through referencing prior studies [7–20], we selected the following variables for possible confounders that may mediate the association of SP with IADL decline: socio-demographics (age, marital status, education, subjective economic status, and work status), health status (body mass index and self-reported chronic medical conditions), lifestyle habits (drinking, smoking, and exercise), and physical and mental functioning (SRH, depression, and cognitive functioning).

#### Socio-economic status

Marital status was categorized as married vs. not married. Educational attainment was categorized as less than 9 years vs. 10 years and more. Regarding subjective economic status, we asked respondents how they felt about their current financial state of affairs, giving them 4 answers to choose from; “very well off,” “somewhat well off,” “somewhat poor” and “poor”. Subjective economic status was dichotomized into good (very/somewhat well off) and poor (i.e., very/somewhat poor). Work status was dichotomized into working (i.e., subjects who had a job with an income at baseline) and not working (i.e., subjects without paid work at baseline).

#### Health status

Body mass index (BMI) was subdivided into underweight (<18.5 kg/m^2^), normal (18.5 to <25.0 kg/m^2^), and overweight (≥25.0 kg/m^2^) categories. Referring to chronic medical conditions, a prior study reported that hypertension, diabetes mellitus, heart disease, and cerebrovascular disease were significantly associated with a decline in IADL [33]. Therefore, we included these four diseases as chronic medical conditions in this study. Subjects were asked if they were currently under medical treatment for hypertension, diabetes mellitus, heart disease, and cerebrovascular disease. Respondents selected “yes” or “no” for each disease.

#### Lifestyle habits

Alcohol consumption was divided into nondrinkers, social drinkers, occasional drinkers, or daily drinkers. Smoking history was categorized as never-smokers, ex-smokers, and current smokers. Regarding exercise, participants were asked about frequency of exercise during the past year; “once a week or more”, “several times a month, “several times a year”, and “almost never”. Subjects who answered ‘‘once a week or more” were defined as exercisers, and those who answered “several times a month or less” were defined as inactive [15].

#### Physical and mental functioning

Self-rated health (SRH) was evaluated by asking a single question “How is your health in general? Is it very good, rather good, rather poor, or very poor?”, and responses dichotomized into good (very/rather good) or poor (very/rather poor). Assessment of depression was conducted using the 5-item short form of the Geriatric Depression Scale [34]; the total score ranges from 0 to 5, with higher scores reflecting a higher level of depression. We defined scores of <2 as subjects without depression, and scores of ≥2 as subjects with depression. Assessment of cognitive functioning was conducted using the MDS Cognitive Performance Scale [35]; total scores range from 0 to 6, with higher scores expressing a lower level of cognitive functioning. The reliability and validity of the Japanese version of the MDS Cognitive Performance Scale have been confirmed [36].We defined a score of 0 as subjects with intact cognitive functioning, and scores of ≥1 as subjects with poor cognitive functioning. We have provided the baseline characteristics of participants by gender in S2 Table.

#### Categorization

All covariates except age were dichotomized: marital status (married vs. unmarried); education (≤9 years of schooling (i.e., low) vs. ≥10 years of schooling); subjective economic status (good vs. poor); work status (working vs. not working); body mass index (normal vs. under/overweight); hypertension (present vs. absent); diabetes mellitus (present vs. absent); heart disease (present vs. absent); cerebrovascular disease (present vs. absent); daily drinkers (yes vs. no); smoking history (ex/current smokers vs. never-smokers); exercise (exercisers vs. inactive); SRH (good vs. poor); depression (present vs. absent); and cognitive functioning (poor vs. intact). Age was categorized as 65–69, 70–74, 75–79, and ≥80. Regarding missing values on the covariates, in accordance with a statistical method for the purpose of coping with missing covariates [37], we performed multiple imputations by chained equations.

### Statistical analysis

We used multiple logistic regression analyses to calculate adjusted odds ratio (OR) and a 95% confidence interval (CI) for IADL decline. The independent variable was the type and frequency of SP at baseline. In Model 1, the age-adjusted OR was calculated. In Model 2, all covariates were added simultaneously, and the multivariable-adjusted OR was calculated. To verify gender differences in the association between the type and frequency of SP and IADL decline, stratified analyses by gender were performed. Additionally, to investigate the effect modification by gender, multiple logistic regression analyses including “gender”, “the frequency of participation in each social activity” and “the interaction term between the frequency of participation and gender” were conducted.

The level of significance was 0.05 (two-tailed). Statistical analyses were conducted using IBM SPSS Statistics (version 24.0; Armonk, New York).

## Results

### Participant characteristics

The cumulative incidence of IADL decline during the 33-month follow-up was significantly higher among men than women (male 16.4% vs. female 8.7%, *P* < 0.001 by chi-squared test). Table 1 shows the baseline characteristics of participants with and without IADL decline by gender. Irrespective of gender, persons with IADL decline were more likely to be older, have less education, have cerebrovascular disease, lead inactive lives, have poorer SRH, feel more depressed, and have poorer cognitive functioning, compared to persons without IADL decline. IADL was not associated with body mass index and smoking history. Men with IADL decline were more likely to be married and have poorer economic status than men without IADL decline. Women with IADL decline were more likely to be unmarried and non-working, have hypertension, diabetes mellitus, and heart disease, and consume less alcohol than women without IADL decline.

**Table 1:** Baseline characteristics of the study population with and without IADL decline according to gender.

Baseline characteristics	Men (n = 2,637)			Women (n = 3,376)		
	No decline	Decline	*P*-value^a^	No decline	Decline	*P*-value^a^
	(n = 2,205)	(n = 432)		(n = 3,081)	(n = 295)	
Age: 75 years and older	30.7%	45.6%	<0.001	30.1%	79.7%	<0.001
Marital status: not married	11.1%	6.3%	0.002	32.7%	46.4%	<0.001
Education (years): ≤9	21.1%	28.2%	0.002	24.5%	41.4%	<0.001
Subjective economic status: poor	53.4%	59.0%	0.035	55.4%	53.6%	0.541
Work status: working	29.4%	25.0%	0.063	15.6%	5.4%	<0.001
Body mass index: underweight	3.4%	2.8%	0.559	8.0%	9.2%	0.503
Body mass index: overweight	21.5%	19.4%	0.367	17.7%	17.6%	1.000
Hypertension: present	40.5%	42.1%	0.556	36.5%	44.1%	0.012
Diabetes mellitus: present	16.7%	15.0%	0.435	8.1%	13.2%	0.004
Heart disease: present	12.8%	12.5%	0.875	7.3%	12.5%	0.002
Cerebrovascular disease: present	3.9%	9.5%	<0.001	1.3%	3.4%	0.011
Alcohol intake: daily drinkers	44.4%	40.7%	0.168	6.8%	3.1%	0.009
Smoking history: ex/current smokers	70.2%	71.5%	0.604	7.9%	7.5%	0.910
Exercise: inactive	56.1%	67.6%	<0.001	56.1%	77.6%	<0.001
Subjects with poor self-rated health	13.3%	20.1%	<0.001	12.8%	33.9%	<0.001
Subjects with depression	15.6%	23.8%	<0.001	19.9%	44.7%	<0.001
Subjects with poor cognitive functioning	14.5%	23.1%	<0.001	11.9%	29.5%	<0.001

IADL, instrumental activities of daily living.

^a^Chi-squared test

### Relationship between the type and frequency of SP and IADL decline

Table 2 shows the adjusted ORs for IADL decline associated with the type and frequency of SP. For men, after age adjustment (Model 1), frequent and moderate participation in hobby clubs, frequent participation in sports groups and senior citizens’ clubs, and moderate participation in neighborhood community associations and cultural clubs were significantly associated with a lower risk of IADL decline, compared to non-participation. After adjustment for all covariates (Model 2), only moderate participation in neighborhood community associations had a protective effect against IADL decline when compared to non-participation (OR = 0.79, 95% CI = 0.63–0.99). For women, after adjustment for age (Model 1), frequent and moderate participation in hobby clubs and cultural clubs, frequent participation in sports groups, and moderate participation in volunteer groups and neighborhood community associations were shown to have a lower risk of IADL decline than non-participation. In Model 2, which adjusted for all covariates, frequent and moderate participation in hobby clubs and moderate participation in volunteer groups, neighborhood community associations, and cultural clubs were significantly associated with a reduced risk of developing IADL decline when compared with non-participation: volunteer groups (moderate participation, OR = 0.53, 95% CI = 0.31–0.88), hobby clubs (frequent participation, OR = 0.63, 95% CI = 0.43–0.93; moderate participation, OR = 0.55, 95% CI = 0.38–0.79), neighborhood community associations (moderate participation, OR = 0.58, 95% CI = 0.42–0.81), and cultural clubs (moderate participation, OR = 0.51, 95% CI = 0.31–0.82).

**Table 2:** Adjusted ORs (95% CIs) for IADL decline associated with the type and frequency of social participation by gender.

Type of SP	Frequency of SP	Men (n = 2,637)				Women (n = 3,376)			
		Model 1^a^		Model 2^b^		Model 1^a^		Model 2^b^	
		OR (95% CI)	*P*-value	OR (95% CI)	*P*-value	OR (95% CI)	*P*-value	OR (95% CI)	*P*-value
Volunteer groups	None	1.00		1.00		1.00		1.00	
	Moderate	0.84 (0.62–1.12)	0.231	0.89 (0.66–1.20)	0.450	0.42 (0.26–0.69)	0.001	0.53 (0.31–0.88)	0.015
	Frequent	0.74 (0.51–1.09)	0.133	0.83 (0.56–1.24)	0.363	0.87 (0.54–1.40)	0.568	1.23 (0.74–2.04)	0.431
Sports groups	None	1.00		1.00		1.00		1.00	
	Moderate	0.80 (0.59–1.08)	0.139	0.89 (0.65–1.21)	0.443	0.55 (0.29–1.02)	0.058	0.65 (0.34–1.26)	0.201
	Frequent	0.68 (0.51–0.89)	0.005	0.86 (0.63–1.17)	0.328	0.42 (0.29–0.60)	<0.001	0.72 (0.48–1.08)	0.111
Hobby clubs	None	1.00		1.00		1.00		1.00	
	Moderate	0.74 (0.58–0.95)	0.016	0.84 (0.65–1.08)	0.168	0.42 (0.30–0.59)	<0.001	0.55 (0.38–0.79)	0.001
	Frequent	0.63 (0.46–0.85)	0.002	0.74 (0.54–1.02)	0.066	0.39 (0.28–0.56)	<0.001	0.63 (0.43–0.93)	0.019
Senior	None	1.00		1.00		1.00		1.00	
citizens’	Moderate	0.89 (0.65–1.21)	0.449	0.96 (0.69–1.32)	0.780	0.76 (0.55–1.05)	0.094	0.83 (0.59–1.17)	0.289
clubs	Frequent	0.53 (0.30–0.93)	0.026	0.62 (0.35–1.11)	0.105	0.72 (0.43–1.18)	0.186	1.07 (0.63–1.80)	0.808
Neighborhood	None	1.00		1.00		1.00		1.00	
community	Moderate	0.74 (0.60–0.92)	0.007	0.79 (0.63–0.99)	0.037	0.48 (0.35–0.66)	<0.001	0.58 (0.42–0.81)	0.001
associations	Frequent	0.66 (0.36–1.18)	0.157	0.64 (0.35–1.17)	0.150	0.60 (0.27–1.33)	0.209	0.98 (0.43–2.26)	0.970
Cultural clubs	None	1.00		1.00		1.00		1.00	
	Moderate	0.61 (0.42–0.86)	0.006	0.71 (0.49–1.02)	0.067	0.39 (0.25–0.62)	<0.001	0.51 (0.31–0.82)	0.006
	Frequent	0.91 (0.50–1.65)	0.757	0.98 (0.53–1.81)	0.936	0.40 (0.21–0.77)	0.006	0.66 (0.33–1.30)	0.231

CI, confidence interval; Frequent, weekly or more; IADL, instrumental activities of daily living; Moderate, monthly or yearly; OR, odds ratio; SP, social participation.

^a^Adjusted for age.

^b^Adjusted for age, marital status, education, subjective economic status, work status, body mass index, hypertension, diabetes mellitus, heart disease, cerebrovascular disease, alcohol, smoking, exercise, self-rated health, depression, and cognitive functioning.

### Comparison between frequent and moderate participation

To draw a comparison between frequent participation and moderate participation, we conducted a subgroup analysis for older adults with participation in each type of SP (Table 3). This additional analysis was based on Model 2, where the data were adjusted for all covariates. Among women, moderate participation in volunteer groups was significantly associated with a decreased risk of IADL decline compared to frequent participation in volunteer groups (OR = 0.37, 95% CI = 0.18–0.77). Among men, for all types of SP, there were no differences between frequent participation and moderate participation in odds of IADL decline.

**Table 3:** Associations of frequent versus moderate participation with IADL decline: Subgroup analyses limited to individuals with participation in each type of SP.

Type of SP	Frequency of SP	Men		Women	
		Model 2^a^		Model 2^a^	
		OR (95% CI)	*P*-value	OR (95% CI)	*P*-value
Volunteer groups	Frequent	1.00		1.00	
	Moderate	1.09 (0.68–1.75)	0.727	0.37 (0.18–0.77)	0.008
Sports groups	Frequent	1.00		1.00	
	Moderate	1.00 (0.66–1.49)	0.980	0.72 (0.33–1.56)	0.404
Hobby clubs	Frequent	1.00		1.00	
	Moderate	1.07 (0.75–1.52)	0.718	0.80 (0.49–1.29)	0.362
Senior citizens’ clubs	Frequent	1.00		1.00	
	Moderate	1.51 (0.78–2.90)	0.223	0.82 (0.45–1.48)	0.509
Neighborhood community associations	Frequent	1.00		1.00	
	Moderate	1.19 (0.64–2.20)	0.589	0.53 (0.21–1.33)	0.175
Cultural clubs	Frequent	1.00		1.00	
	Moderate	0.77 (0.37–1.60)	0.480	0.84 (0.36–1.99)	0.697

CI, confidence interval; Frequent, weekly or more; IADL, instrumental activities of daily living; Moderate, monthly or yearly; OR, odds ratio; SP, social participation.

^a^Adjusted for age, marital status, education, subjective economic status, work status, body mass index, hypertension, diabetes mellitus, heart disease, cerebrovascular disease, alcohol, smoking, exercise, self-rated health, depression, and cognitive functioning.

### Interaction effect between SP and gender on IADL decline

The results of effect modification by gender on the association between the type and frequency of SP and IADL decline in Model 2 with interaction terms are provided in S3 Table. Significant interactions between moderate participation and gender were observed in volunteer groups (OR = 0.53, 95% CI = 0.30–0.95), hobby clubs (OR = 0.57, 95% CI = 0.37–0.87), and neighborhood community associations (OR = 0.63, 95% CI = 0.43–0.92). Significant interaction between frequent participation and gender was observed only in senior citizens’ clubs (OR = 2.31, 95% CI = 1.08–4.92).

**Fig 2:**
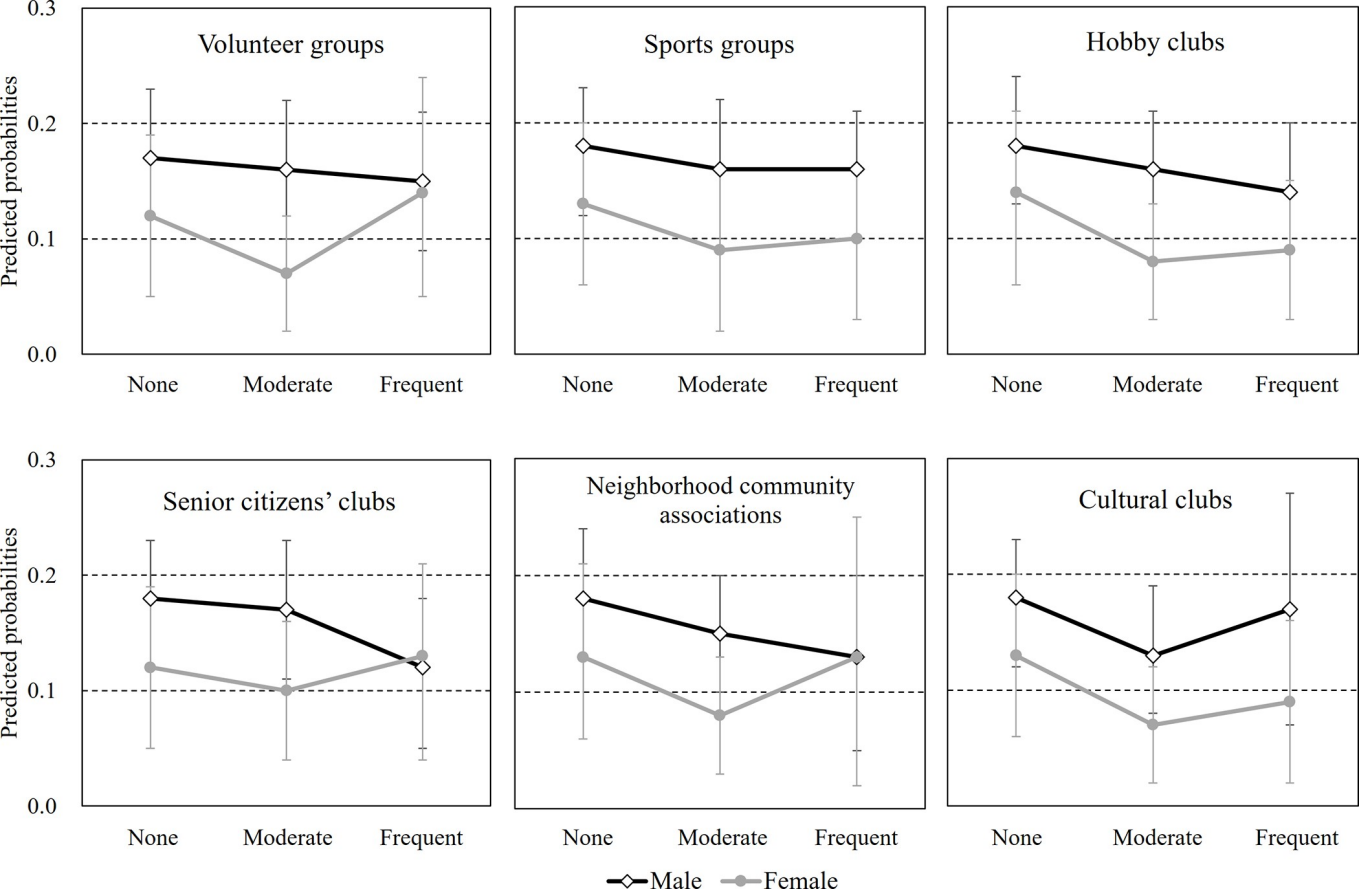
Predicted probabilities for IADL decline according to gender and the frequency of SP. Error bars display 95% confidence intervals. Dashed horizontal lines indicate scale marks for the y-axis; Predicted probabilities are estimated based on the logistic regression which was adjusted for all covariates such as age, marital status, education, subjective economic status, work status, body mass index, hypertension, diabetes mellitus, heart disease, cerebrovascular disease, alcohol, smoking, exercise, SRH, depression, and cognitive functioning.

Fig 2 displays the plot of predicted probabilities for IADL decline. As a whole, men tended to have a higher probability of IADL decline compared with women, regardless of the types and frequency for SP. In contrast, for volunteer groups, senior citizens’ clubs, and neighborhood community associations, men were less likely to have a probability of IADL decline with more frequent participation, whereas women with frequent participation had a higher probability of IADL decline than women with moderate participation, showing that frequent participation had no gender difference in a probability of IADL decline.

### Additional stratified analyses by SRH, depression, and cognitive functioning

Because not only gender, but also physical and mental functioning can have a great influence on IADL decline in older people [25,31], we performed further stratified analyses according to SRH (i.e., good or poor) [38], depression (i.e., absent or present) [18, 39], and cognitive functioning (i.e., intact or poor) [20,40]; the results are shown in S4–S6 Tables. Significant associations between moderate participation in volunteer groups and a lower risk of IADL decline were observed only in participants with depression or those with poor cognitive functioning. Regarding sports groups, significant associations between frequent participation and IADL maintenance were observed only in individuals with good SRH or those with depression. Significant relationships between frequent and moderate participation in hobby clubs and IADL maintenance were observed among people with good SRH but not among those with poor SRH, while they were not affected by the presence or absence of depression. Regarding neighborhood community associations, moderate participation was associated with significantly lower odds of IADL decline only among people with good SRH or those with intact cognition. In contrast, a significant association between moderate participation in cultural clubs and IADL maintenance was little affected by physical and mental functioning.

## Discussion

This study showed that in some types of SP, moderate rather than frequent participation was more effective than non-participation, and that in models limited to people participating in each type of SP, women with moderate participation in volunteer groups had a significantly lower risk of IADL decline than women with frequent participation in volunteer groups. Additionally, the results of the effect modification by gender indicated that women were more likely than men to have a beneficial effect on IADL by moderate participation in volunteer groups, hobby clubs, and neighborhood community associations, while men were more likely to benefit from frequent participation in senior citizens’ clubs than women. Also, additional stratified analyses suggested that physical and mental functioning played a great role of the type and frequency of SP on IADL maintenance.

Some researchers have argued that SP can have both positive and negative influences on older adults’ health [41,42]. On the negative side, SP has the potential to cause human-relation problems, be an emotional burden, and give rise to harmful behaviours. Our previous studies have indicated that obligatory SP has no positive impact on the health of older adults and occasionally results in poor health effects [28,43]. In the present study, moderate participation in neighborhood community associations was more favorable for the maintenance of IADL among women than men. Because people often participate in neighborhood community associations involuntarily [28], frequent involvement in obligatory SP may impose a mental burden on participants, leading to limited psychological benefits from SP. Negative psychological states are reported to be an independent risk factor for IADL decline among high-functioning older people [44]. Additionally, a prior study of community-dwelling older people reported that SP involving contact with neighbors was significantly associated with a reduced risk of psychological distress in older women, but not in older men [7]. Where the frequency in participation is monthly or yearly, participation in neighborhood community associations may promote community-based interaction and offer more preventive effects against IADL decline for older women than older men.

In relation to hobby clubs, women with frequent and moderate participation were more likely to preserve IADL ability than women without participation, and women derived more benefit from moderate participation than men. Hobby clubs bring together people who wish to pursue with others a shared interest in a particular hobby. Because participating in hobby clubs is about doing what one loves, frequent participation in hobby clubs is quite unlikely to place psychological stress on participants. Furthermore, women are more sensitive to closeness with the members of their network than men, and the intimacy of human relationships has a greater effect on health for women than for men [45,46]. Because members of hobby clubs share a common interest, they are more likely to open up to one another and achieve an intimate relationship than those involved in other types of SP. Moderate participation in hobby clubs may provide more women than men with close relationships and affect female IADL in a positive way.

Regarding volunteer groups, only women who participated moderately had a significant association with lower risk of IADL decline. This finding is consistent with a prior study which demonstrated that a positive effect of volunteering on longevity was exerted by moderate participation, but not by frequent participation [17]. However, this study [17] examined neither gender-specific health effects of volunteer activities nor the association of frequent versus moderate participation with health outcome. The present study has offered new evidence that moderate participation in volunteer groups is more beneficial to women than to men, and that among women, moderate participation is significantly associated with a lower risk of IADL decline than frequent participation. Regarding the difference between volunteer groups and other types of SP, involvement in volunteer groups requires caring more about those who receive support from a volunteer than about him/herself; that is, volunteering has an aspect of self-sacrifice [47]. On the negative side, volunteering can cause a heavier psychological burden due to frequency of activities and pressure to increase participation [17]. This could be why repeated volunteer activities do not appear to offer good effects on health. Because women particularly have a greater tendency than men to put everyone else's needs before their own [45,48], frequent participation in volunteer groups may generate a stronger negative effect on females than on men. On the other hand, volunteer activities provide a positive aspect for participants as they feel they are useful to others [47]. The employment rate of adult Japanese women is lower than in most other OECD countries and many of them are non-regular workers [49]. Therefore, Japanese women have fewer opportunities to acquire social roles through work in their adult life than males. Participating in volunteer activities in their latter life may give women the opportunity to feel they are contributing to society. Thus, because high-frequency volunteer activities are highly likely to impose an inordinate burden on females, the good effect on IADL of participation in volunteer groups may be experienced by women who only do so in low frequency, resulting in a significant difference between moderate participation and frequent participation in volunteer groups among women.

For frequent participation in senior citizens’ clubs, men saw more beneficial effects on IADL performance than women. Recently, researchers in public health have given attention to social capital as a potential approach to health promotion [50]; bonding social capital refers to connections among homogenous people who are similar with respect to socio-demographic characteristics such as age, ethnicity, and social class, while bridging social capital is derived from connections among individuals who are dissimilar. Senior citizens’ clubs have age limits, such as for persons 60 years of age or older, whereas other types of SP than senior citizens’ clubs are open for all ages. That is, participation in senior citizens’ clubs is regarded as a property of bonding social capital. A prior population-based study in Japan examined the association between bonding versus bridging social capital and self-rated health, and demonstrated that men had more health benefits from bonding social capital than women [51]. Our previous study has showed that SRH is a strong predictor of IADL decline among community-dwelling older adults [38]. Therefore, men who frequently participate in senior citizens’ clubs may tend to keep their SRH in good condition compare to women with frequent participation, which in turn generates a positive impact on IADL ability for men.

Our results based on additional stratified analyses have suggested the following; 1) Moderate participation in volunteer groups may have a great protective effect on IADL decline among old people with poor mental health, such as depression and poor cognitive functioning, 2) Moderate participation in neighborhood community associations and frequent and moderate participation in hobby clubs may make a major contribution to the prevention of IADL decline among old people with good physical and mental functioning, such as good SRH and intact cognition, and 3) Moderate participation in cultural clubs may result in better IADL ability, regardless of physical and mental functioning. These results agree with those of previous studies among community-dwelling older adults, that physical and mental functioning can have a potent influence on the association between SP in older ages and IADL [23–26,52,53]. Our findings suggest that a positive effect of SP on IADL ability varies depending on not only gender and the type and frequency of SP, but also physical and mental functioning.

Our study has several limitations. First, we did not succeed in achieving adequate response and follow-up rates. As shown in S1 Table, individuals who were excluded from this study tended to have poorer SRH, more depressive symptoms, and poorer cognitive functioning than analyzed persons. Because deterioration in physical and mental functioning can increase the risk of developing IADL decline [25,31,38–40], there is a strong possibility that excluded people were at high-risk for IADL decline. This bias may act to underestimate the association between SP and IADL. Second, this study is a prospective cohort study, but has the potential for reverse causation. Being reluctant to participate in social activities may be a premonitory symptom of IADL decline [14,20]. Third, although we could evaluate IADL at both baseline and follow-up, the assessment of SP was done at baseline alone; this study failed to assess the change in SP status. Because a change in participation in social activities can affect the mental health [53,54] and functional capacity [53] of older adults, further studies are needed to ascertain the association of transitions in SP with changes in IADL. Fourth, both the independent variable (i.e., SP) and the dependent variable (i.e., IADL) were evaluated by self-report; observed associations may be overestimated by same-source bias [55]. Finally, the study population of this study comprised older adults who had independent IADL at baseline and were living in a commuter town in Japan. Because IADL performance is affected by the public transport system in the neighborhood and gender roles [22,56], we should take great care when generalizing our findings to older adults with functional disabilities and people living in a rural area or in a country with a different cultural background.

## Conclusions

This study examined the relationship between incident decline in IADL and SP from the perspective of type of social activities and frequency of participation in a community-based prospective cohort study. Our results have demonstrated that moderate SP rather than frequent SP may produce beneficial effects in the prevention of IADL decline and that regarding volunteer groups, women with moderate participation may see a more beneficial effect on IADL than women with frequent participation. Additionally, the effective types of SP against IADL decline are different in gender and physical and mental functioning. Therefore, when public health nurses and family physicians recommend SP to aged people with the purpose of preventing IADL decline, it is necessary to consider their gender and physical and mental functioning, and at the same time, to refrain from asking them for frequent participation in certain types of SP.

## Supporting information

S1 Table Basic attributes of analyzed participants and subjects excluded due to missing data or loss to follow-up. (PDF)

S2 Table Baseline characteristics of study participants by gender. (PDF)

S3 Table Adjusted ORs (95% CIs) for IADL decline by participation in each group or club type: results of the effect modification of the type and frequency of SP and gender (n = 6,013). (PDF)

S4 Table Adjusted ORs (95% CIs) for IADL decline with the type and frequency of SP based on stratified analyses by self-rated health. (DOCX)

S5 Table Adjusted ORs (95% CIs) for IADL decline with the type and frequency of SP based on stratified analyses by depression. (DOCX)

S6 Table Adjusted ORs (95% CIs) for IADL decline with the type and frequency of SP based on stratified analyses by cognitive functioning. (DOCX)

S1 File De-identified dataset used for the statistical analyses. (XLSX)
