# Supplementary material for: The differential effects of type and frequency of social participation on IADL declines of older people
Source: PLoS One. 2018 Nov 21;13(11):e0207426. doi: 10.1371/journal.pone.0207426 (PMC6248949; doi:10.1371/journal.pone.0207426)
Supplement: S1 Table — (PDF) [file pone.0207426.s001.pdf]

S1 Table. Basic attributes of analyzed participants and subjects excluded due to missing data or lost to follow-up

	Analyzed subjects (n = 6,013)		Excluded subjects (n = 2,919)		*P*-value[a]
	n	(%)	n	(%)	
Gender: males	2,637	(43.9)	1,280	(43.9)	1.000
Age: 75 years or older	2,034	(33.8)	1,294	(44.3)	<0.001
Subjects with poor self-rated health	874	(14.5)	553	(18.9)	<0.001
Subjects with depression	1,194	(19.9)	714	(24.5)	<0.001
Subjects with poor cognitive functioning	873	(14.5)	529	(18.1)	<0.001

[a] Differences between the two groups were analyzed using chi-squared test.
