# Supplementary material for: The differential effects of type and frequency of social participation on IADL declines of older people
Source: PLoS One. 2018 Nov 21;13(11):e0207426. doi: 10.1371/journal.pone.0207426 (PMC6248949; doi:10.1371/journal.pone.0207426)
Supplement: S2 Table — (PDF) [file pone.0207426.s002.pdf]

S2 Table. Baseline characteristics of study participants by gender

	Men (n = 2,637)		Women (n = 3,376)		*P*-value[a]
	n	(%)	n	(%)	
**Socio-demographics**					
Age at baseline survey (years)					0.070
65-69	894	(33.9)	1,153	(34.2)	
70-74	870	(33.0)	1,062	(31.5)	
75-79	529	(20.1)	645	(19.1)	
≥80	344	(13.0)	516	(15.3)	
Marital status					<0.001
Currently married	2,334	(88.5)	2,161	(64.0)	
Not married	271	(10.3)	1,143	(33.9)	
Missing data	32	(1.2)	72	(2.1)	
Education (years of schooling)					
High (≥13)	838	(31.8)	589	(17.4)	<0.001
Medium (10-12)	1,193	(45.2)	1,872	(55.5)	
Low (≤9)	588	(22.3)	878	(26.0)	
Missing data	18	(0.7)	37	(1.1)	
Subjective economic status					0.113
Very poor	377	(14.3)	530	(15.7)	
Somewhat poor	1,055	(40.0)	1,335	(39.5)	
Somewhat well off	909	(34.5)	1,081	(32.0)	
Very well off	110	(4.2)	162	(4.8)	
Missing data	186	(7.1)	268	(7.9)	
Work status					<0.001
Working	757	(28.7)	496	(14.7)	
Not working	1,880	(71.3)	2,880	(85.3)	
**Health status**					
Body mass index					<0.001
Normal	1,902	(72.1)	2,364	(70.0)	
Underweight	88	(3.3)	274	(8.1)	
Overweight	557	(21.1)	597	(17.7)	
Missing data	90	(3.4)	141	(4.2)	
Chronic medical conditions (subjects with each disease or with missing data)					
Hypertension	1,076	(40.8)	1,254	(37.1)	0.004
Diabetes mellitus	433	(16.4)	289	(8.6)	<0.001
Heart disease	337	(12.8)	262	(7.8)	<0.001
Cerebrovascular disease	127	(4.8)	51	(1.5)	<0.001
Missing data	225	(8.5)	347	(10.3)	0.024

S2 Table. Continued

	Men (n = 2,637)		Women (n = 3,376)		*P*-value[a]
	n	(%)	n	(%)	
**Lifestyle habits**					
Alcohol intake					<0.001
Non-drinkers	385	(14.6)	1,642	(48.6)	
Social drinkers	506	(19.2)	921	(27.3)	
Occasional drinkers	458	(17.4)	448	(13.3)	
Daily drinkers	1,154	(43.8)	218	(6.5)	
Missing data	134	(5.1)	147	(4.4)	
Smoking history					<0.001
Never-smokers	647	(24.5)	2,939	(87.1)	
Ex-smokers	1,434	(54.4)	176	(5.2)	
Current smokers	423	(16.0)	89	(2.6)	
Missing data	133	(5.0)	172	(5.1)	
Frequency of exercise					<0.001
Once a week or more	1,021	(38.7)	1,219	(36.1)	
Several times a month	347	(13.2)	307	(9.1)	
Several times a year	134	(5.1)	85	(2.5)	
Almost never	1,049	(39.8)	1,564	(46.3)	
Missing data	86	(3.3)	201	(6.0)	
**Physical and mental functioning**					
Self-rated health					0.102
Good	2,079	(78.8)	2,699	(79.9)	
Poor	380	(14.4)	494	(14.6)	
Missing data	178	(6.8)	183	(5.4)	
Depression (score of the Geriatric Depression Scale)					<0.001
No depression (<2)	2,039	(77.3)	2,455	(72.7)	
Depression (≥2)	448	(17.0)	746	(22.1)	
Missing data	150	(5.7)	175	(5.2)	
Cognitive functioning (score of the Cognitive Performance Scale)					0.018
Intact (0)	2,172	(82.4)	2,872	(85.1)	
Poor (≥1)	419	(15.9)	454	(13.4)	
Missing data	46	(1.7)	50	(1.5)	

S2 Table. Continued

	Men (n = 2,637)		Women (n = 3,376)		*P*-value[a]
	n	(%)	n	(%)	
**Type and frequency of social participation**					
Participation in volunteer groups					0.011
None	1,958	(74.3)	2,619	(77.6)	
Moderate	432	(16.4)	481	(14.2)	
Frequent	247	(9.4)	276	(8.2)	
Participation in sports groups					<0.001
None	1,618	(61.4)	2,203	(65.3)	
Moderate	446	(16.9)	223	(6.6)	
Frequent	573	(21.7)	950	(28.1)	
Participation in hobby clubs					<0.001
None	1,419	(53.8)	1,669	(49.4)	
Moderate	749	(28.4)	865	(25.6)	
Frequent	469	(17.8)	842	(24.9)	
Participation in senior citizens’ clubs					<0.001
None	2,193	(83.2)	2,649	(78.5)	
Moderate	321	(12.2)	535	(15.8)	
Frequent	123	(4.7)	192	(5.7)	
Participation in neighborhood community associations					<0.001
None	1,405	(53.3)	2,071	(61.3)	
Moderate	1,129	(42.8)	1,233	(36.5)	
Frequent	103	(3.9)	72	(2.1)	
Participation in cultural clubs					<0.001
None	2,222	(84.3)	2,610	(77.3)	
Moderate	329	(12.5)	545	(16.1)	
Frequent	86	(3.3)	221	(6.5)	

Frequent, weekly or more; Moderate, monthly or yearly.

[a] Differences between men and women were analyzed using chi-squared test.
