# Supplementary material for: The differential effects of type and frequency of social participation on IADL declines of older people
Source: PLoS One. 2018 Nov 21;13(11):e0207426. doi: 10.1371/journal.pone.0207426 (PMC6248949; doi:10.1371/journal.pone.0207426)
Supplement: S3 Table — (PDF) [file pone.0207426.s003.pdf]

S3 Table. Adjusted ORs (95% CIs) for IADL decline by participation in each group or club type: results of the effect modification of the type and frequency of SP and gender (n = 6,013)

Type of SP	Model 2 with interaction terms	
	OR[a] (95% CI)	*P*-value
Participation in volunteer groups		
Gender men	1.00	
Gender women	0.53 (0.41-0.69)	<0.001
None	1.00	
Moderate	0.92 (0.67-1.25)	0.581
Frequent	0.89 (0.60-1.34)	0.584
None × gender	1.00	
Moderate × gender	0.53 (0.30-0.95)	0.032
Frequent × gender	1.19 (0.64-2.20)	0.581
Participation in sports groups		
Gender men	1.00	
Gender women	0.57 (0.43-0.74)	<0.001
None	1.00	
Moderate	0.96 (0.70-1.32)	0.811
Frequent	0.88 (0.65-1.20)	0.416
None × gender	1.00	
Moderate × gender	0.58 (0.29-1.16)	0.122
Frequent × gender	0.65 (0.41-1.03)	0.069
Participation in hobby clubs		
Gender men	1.00	
Gender women	0.61 (0.46-0.80)	<0.001
None	1.00	
Moderate	0.88 (0.68-1.15)	0.356
Frequent	0.77 (0.55-1.06)	0.102
None × gender	1.00	
Moderate × gender	0.57 (0.37-0.87)	0.010
Frequent × gender	0.69 (0.43-1.11)	0.124

S3 Table. Continued.

Type of SP	Model 2 with interaction terms	
	OR[a] (95% CI)	*P*-value
Participation in senior citizens' clubs		
Gender men	1.00	
Gender women	0.47 (0.36-0.61)	<0.001
None	1.00	
Moderate	0.77 (0.55-1.07)	0.113
Frequent	0.50 (0.28-0.90)	0.021
None × gender	1.00	
Moderate × gender	1.38 (0.88-2.17)	0.157
Frequent × gender	2.31 (1.08-4.92)	0.031
Participation in neighborhood community associations		
Gender men	1.00	
Gender women	0.55 (0.42-0.73)	<0.001
None	1.00	
Moderate	0.81 (0.65-1.02)	0.072
Frequent	0.63 (0.34-1.17)	0.148
None × gender	1.00	
Moderate × gender	0.63 (0.43-0.92)	0.018
Frequent × gender	1.61 (0.60-4.36)	0.345
Participation in cultural clubs		
Gender men	1.00	
Gender women	0.54 (0.42-0.70)	<0.001
None	1.00	
Moderate	0.71 (0.49-1.03)	0.068
Frequent	1.02 (0.54-1.93)	0.942
None × gender	1.00	
Moderate × gender	0.67 (0.37-1.22)	0.190
Frequent × gender	0.58 (0.23-1.41)	0.228

CI, confidence interval; Frequent, weekly or more; IADL, instrumental activities of daily living; Moderate, monthly or yearly; OR, odds ratio; SP, social participation.

[a]Adjusted for gender, age, marital status, education, subjective economic status, work status, body mass index, hypertension, diabetes mellitus, heart disease, cerebrovascular disease, alcohol, smoking, exercise, self-rated health, depression, and cognitive functioning.
