# Supplementary material for: The differential effects of type and frequency of social participation on IADL declines of older people
Source: PLoS One. 2018 Nov 21;13(11):e0207426. doi: 10.1371/journal.pone.0207426 (PMC6248949; doi:10.1371/journal.pone.0207426)
Supplement: S4 Table — (DOCX) [file pone.0207426.s004.docx]

**S4 Table. Adjusted ORs (95% CIs) for IADL decline with the type and frequency of SP based on stratified analyses by self-rated health**

| Type  of SP | Frequency  of SP | Self-rated health | | | | |
| --- | --- | --- | --- | --- | --- | --- |
|  |  | Good (n = 5,139)^a^ | |  | Poor (n = 874)^a^ | |
|  |  | OR^b^ (95% CI) | *P*-value |  | OR^b^ (95% CI) | *P*-value |
| Volunteer  groups | None | 1.00 |  |  | 1.00 |  |
|  | Moderate | 0.78 (0.58-1.03) | 0.081 |  | 0.58 (0.31-1.11) | 0.102 |
|  | Frequent | 1.00 (0.72-1.40) | 0.990 |  | 0.79 (0.35-1.80) | 0.572 |
| Sports  groups | None | 1.00 |  |  | 1.00 |  |
|  | Moderate | 0.81 (0.60-1.10) | 0.176 |  | 0.86 (0.42-1.76) | 0.685 |
|  | Frequent | 0.71 (0.54-0.94) | 0.016 |  | 0.87 (0.50-1.53) | 0.626 |
| Hobby  clubs | None | 1.00 |  |  | 1.00 |  |
|  | Moderate | 0.71 (0.56-0.90) | 0.004 |  | 0.70 (0.44-1.11) | 0.125 |
|  | Frequent | 0.63 (0.48-0.83) | 0.001 |  | 0.77 (0.44-1.35) | 0.366 |
| Senior  citizens’ clubs | None | 1.00 |  |  | 1.00 |  |
|  | Moderate | 0.94 (0.72-1.23) | 0.660 |  | 0.80 (0.48-1.31) | 0.372 |
|  | Frequent | 0.80 (0.52-1.21) | 0.285 |  | 0.70 (0.25-1.94) | 0.486 |
| Neighborhood  community associations | None | 1.00 |  |  | 1.00 |  |
|  | Moderate | 0.67 (0.54-0.82) | <0.001 |  | 0.75 (0.51-1.10) | 0.140 |
|  | Frequent | 0.79 (0.47-1.32) | 0.368 |  | 0.43 (0.09-2.10) | 0.298 |
| Cultural  clubs | None | 1.00 |  |  | 1.00 |  |
|  | Moderate | 0.63 (0.45-0.86) | 0.004 |  | 0.48 (0.25-0.95) | 0.034 |
|  | Frequent | 0.82 (0.50-1.32) | 0.406 |  | 0.55 (0.15-2.02) | 0.364 |

CI, confidence interval; Frequent, weekly or more; IADL, instrumental activities of daily living; Moderate, monthly or yearly; OR, odds ratio; SP, social participation.

^a^The pooled number by multiple imputations.

^b^Adjusted for gender, age, marital status, education, subjective economic status, work status, body mass index, hypertension, diabetes mellitus, heart disease, cerebrovascular disease, alcohol, smoking, exercise, depression, and cognitive functioning.
