# Supplementary material for: The differential effects of type and frequency of social participation on IADL declines of older people
Source: PLoS One. 2018 Nov 21;13(11):e0207426. doi: 10.1371/journal.pone.0207426 (PMC6248949; doi:10.1371/journal.pone.0207426)
Supplement: S5 Table — (DOCX) [file pone.0207426.s005.docx]

**S5 Table.** **Adjusted ORs (95% CIs) for IADL decline with the type and frequency of SP based on stratified analyses by depression**

| Type  of SP | Frequency  of SP | Depression | | | | |
| --- | --- | --- | --- | --- | --- | --- |
|  |  | Absent (n = 4,737)^a^ | |  | Present (n = 1,276)^a^ | |
|  |  | OR^b^ (95% CI) | *P*-value |  | OR^b^ (95% CI) | *P*-value |
| Volunteer  groups | None | 1.00 |  |  | 1.00 |  |
|  | Moderate | 0.83 (0.62-1.11) | 0.216 |  | 0.50 (0.29-0.87) | 0.015 |
|  | Frequent | 0.88 (0.60-1.29) | 0.521 |  | 1.21 (0.68-2.16) | 0.511 |
| Sports  groups | None | 1.00 |  |  | 1.00 |  |
|  | Moderate | 0.90 (0.66-1.24) | 0.526 |  | 0.59 (0.31-1.14) | 0.116 |
|  | Frequent | 0.82 (0.62-1.10) | 0.189 |  | 0.55 (0.33-0.93) | 0.024 |
| Hobby  clubs | None | 1.00 |  |  | 1.00 |  |
|  | Moderate | 0.71 (0.55-0.92) | 0.010 |  | 0.66 (0.44-1.01) | 0.053 |
|  | Frequent | 0.67 (0.50-0.89) | 0.005 |  | 0.61 (0.38-0.98) | 0.041 |
| Senior  citizens’ clubs | None | 1.00 |  |  | 1.00 |  |
|  | Moderate | 0.96 (0.72-1.28) | 0.769 |  | 0.76 (0.51-1.14) | 0.189 |
|  | Frequent | 0.76 (0.46-1.26) | 0.281 |  | 0.81 (0.38-1.72) | 0.582 |
| Neighborhood  community associations | None | 1.00 |  |  | 1.00 |  |
|  | Moderate | 0.70 (0.56-0.87) | 0.001 |  | 0.67 (0.47-0.94) | 0.021 |
|  | Frequent | 0.91 (0.52-1.59) | 0.727 |  | 0.35 (0.10-1.23) | 0.101 |
| Cultural  clubs | None | 1.00 |  |  | 1.00 |  |
|  | Moderate | 0.64 (0.46-0.89) | 0.009 |  | 0.51 (0.27-0.95) | 0.032 |
|  | Frequent | 0.96 (0.59-1.58) | 0.878 |  | 0.38 (0.12-1.16) | 0.089 |

CI, confidence interval; Frequent, weekly or more; IADL, instrumental activities of daily living; Moderate, monthly or yearly; OR, odds ratio; SP, social participation.

^a^The pooled number by multiple imputations.

^b^Adjusted for gender, age, marital status, education, subjective economic status, work status, body mass index, hypertension, diabetes mellitus, heart disease, cerebrovascular disease, alcohol, smoking, exercise, self-rated health, and cognitive functioning.
