# Supplementary material for: The differential effects of type and frequency of social participation on IADL declines of older people
Source: PLoS One. 2018 Nov 21;13(11):e0207426. doi: 10.1371/journal.pone.0207426 (PMC6248949; doi:10.1371/journal.pone.0207426)
Supplement: S6 Table — (DOCX) [file pone.0207426.s006.docx]

**S6 Table. Adjusted ORs (95% CIs) for IADL decline with the type and frequency of SP based on stratified analyses by cognitive functioning**

| Type  of SP | Frequency  of SP | Cognitive functioning | | | |
| --- | --- | --- | --- | --- | --- |
|  |  | Intact (n = 5,123)^a^ | | Poor (n =890)^a^ | |
|  |  | OR^b^ (95% CI) | *P*-value | OR^b^ (95% CI) | *P*-value |
| Volunteer  groups | None | 1.00 |  | 1.00 |  |
|  | Moderate | 0.82 (0.61-1.10) | 0.178 | 0.56 (0.32-0.98) | 0.041 |
|  | Frequent | 1.07 (0.76-1.50) | 0.717 | 0.70 (0.34-1.43) | 0.331 |
| Sports  groups | None | 1.00 |  | 1.00 |  |
|  | Moderate | 0.92 (0.68-1.25) | 0.594 | 0.57 (0.30-1.06) | 0.076 |
|  | Frequent | 0.77 (0.58-1.01) | 0.062 | 0.62 (0.36-1.05) | 0.074 |
| Hobby  clubs | None | 1.00 |  | 1.00 |  |
|  | Moderate | 0.70 (0.56-0.89) | 0.003 | 0.73 (0.46-1.15) | 0.173 |
|  | Frequent | 0.71 (0.54-0.93) | 0.012 | 0.46 (0.26-0.82) | 0.008 |
| Senior  citizens’ clubs | None | 1.00 |  | 1.00 |  |
|  | Moderate | 0.99 (0.76-1.30) | 0.954 | 0.64 (0.39-1.05) | 0.079 |
|  | Frequent | 0.83 (0.53-1.30) | 0.416 | 0.68 (0.32-1.43) | 0.306 |
| Neighborhood  community associations | None | 1.00 |  | 1.00 |  |
|  | Moderate | 0.64 (0.52-0.78) | <0.001 | 0.84 (0.58-1.23) | 0.380 |
|  | Frequent | 0.75 (0.43-1.31) | 0.311 | 0.73 (0.25-2.11) | 0.564 |
| Cultural  clubs | None | 1.00 |  | 1.00 |  |
|  | Moderate | 0.61 (0.44-0.84) | 0.003 | 0.54 (0.28-1.06) | 0.072 |
|  | Frequent | 0.86 (0.52-1.42) | 0.548 | 0.69 (0.26-1.84) | 0.454 |

CI, confidence interval; Frequent, weekly or more; IADL, instrumental activities of daily living; Moderate, monthly or yearly; OR, odds ratio; SP, social participation.

^a^The pooled number by multiple imputations.

^b^Adjusted for gender, age, marital status, education, subjective economic status, work status, body mass index, hypertension, diabetes mellitus, heart disease, cerebrovascular disease, alcohol, smoking, exercise, self-rated health, and depression.
